# The Prevalence of Syphilis Infection and Its Associated Factors in the General Population of Rwanda: A National Household-Based Survey

**DOI:** 10.1155/2016/4980417

**Published:** 2016-03-30

**Authors:** Mwumvaneza Mutagoma, Eric Remera, Dieudonné Sebuhoro, Steve Kanters, David J. Riedel, Sabin Nsanzimana

**Affiliations:** ^1^Rwanda Biomedical Center, Ministry of Health, Kigali, Rwanda; ^2^Global Evaluative Sciences, Vancouver, BC, Canada; ^3^School of Population and Public Health, University of British Columbia, Vancouver, BC, Canada; ^4^Institute of Human Virology and Division of Infectious Diseases, University of Maryland School of Medicine, Baltimore, MD, USA; ^5^Basel Clinical Epidemiology and Biostatistics Institute & Swiss Tropical and Public Health Institute, University of Basel, Basel, Switzerland

## Abstract

*Background*. The prevalence of syphilis among HIV-infected people is a public health concern, but there is limited literature to describe the true burden of syphilis in resource-limited settings. We conducted this survey in 2013 to estimate the prevalence of syphilis.* Methods*. A cross-sectional survey. Participants were tested for syphilis and HIV. Factors associated with syphilis infection were identified.* Results*. The prevalence of syphilis was 0.9% (95% CI: 0.7–1.1). This prevalence was higher in the 25–49-year-old age (1.1% [95% CI: 0.8–1.3]) than in the 15–24-year-old age (0.6% (95% CI: 0.4–0.9)). Women with lower education had a higher prevalence of syphilis (1.2% (95% CI: 0.9–1.5)) compared to others (0.4% (95% CI: 0.2–0.8)). This prevalence among HIV-infected people was six times higher: 4.8% (95% CI: 2.9–7.9) compared to HIV-negative people (0.8% (95% CI: 0.6–1.0)). The prevalence of syphilis among HIV-infected females was 5.9% (95% CI: 3.4–10.0). HIV-infected or concurrent sexual partners was associated with increased syphilis prevalence with aOR = 4.2 (95% CI: 2.5–7.2) and aOR = 4.2 (95% CI: 2.8–6.5), respectively.* Conclusions*. The prevalence of syphilis was significantly higher among HIV-infected patients. HIV infection and concurrent sexual partners are associated with an increased prevalence of syphilis. Preventing HIV might help in preventing syphilis.

## 1. Introduction

Syphilis is an ulcerative sexually transmitted infection (STI) that remains a major global public health problem. In 2008, the World Health Organization (WHO) estimated that 36.4 million people were infected with syphilis worldwide [1]. It is estimated that there are more than 12 million new syphilis infections every year in the world [2, 3], of which 90% of cases are found in resource-limited countries [4]. The WHO reported that the annual new cases of syphilis in the African region among adults aged 15–49 was 3.4 million [1].

Syphilis infection remains an important STI due to its prevalence, infectiousness, and toll on both infected individuals and health systems. Its importance is exacerbated by the fact that its ulcerative lesions facilitate HIV acquisition; another important aspect of syphilis is its potential vertical transmission [5–7]. The risk of acquiring HIV infection through sexual intercourse is increased 3–5 times in individuals who are infected with syphilis compared to individuals who are not [8]. Primary and secondary syphilis rates have increased every year in United States due to HIV pandemic [8, 9]. This association was also found in neighboring Kenya where prevalence of syphilis in a national survey was higher among HIV-infected persons (6.4%) compared to HIV-negative individuals (1.6%) [10].

Until now there has not been a representative country survey of syphilis in Rwanda. The only estimates of syphilis were studied in specific groups that were not nationally representative with increased risk for syphilis infection, such as pregnant women, female sex workers, and patients attending health facilities seeking STDs treatment. In samples of pregnant women attending antenatal clinic, the prevalence of syphilis was 2.5% in 2007 and 2.0% in 2011 [11]. The aim of this study was to determine the prevalence of syphilis infection and its risk factors in the general population of Rwanda. Results from this study will be important to inform national and public health policy and to determine specific interventions necessary to curb the spread of this disease.

## 2. Methods

### 2.1. Study Design

**Figure 1:**
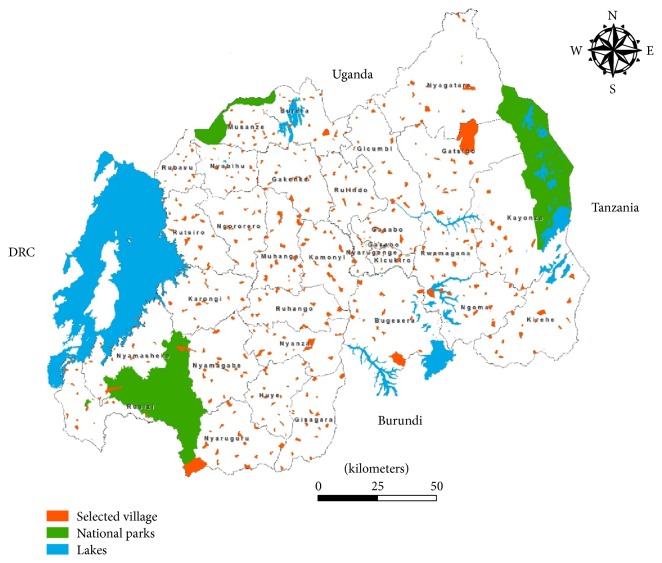
Selected villages in AIDS indicator and HIV incidence survey, Rwanda 2013.

Between June 2013 and November 2013, we conducted a cross-sectional, nationwide, population-based survey. The study was designed for AIDS indicator and HIV incidence survey. The target population included all women and men aged 15–49 living in Rwanda. The study was designed to allow reliable estimation of variables relating to knowledge and behaviour regarding STIs and HIV and the prevalence of syphilis infection. We used the same sampling frame as 2010 Rwanda Demographic Health Survey (RDHS) [12]. The sample was selected in two cluster stages. In the first stage, 492 villages were randomly selected in all districts of Rwanda. A complete mapping and listing of all households existing in the selected villages was conducted. The list of villages served as the sampling frame for the second stage of sample selection. Households were systematically numbered and listed for participation in the survey. The sampling population included all 30 districts, 416 sectors, and 14,837 villages (Figure 1).

Based on the 2010 RDHS, we expected an average of two adults in the specified age groups per household. The survey was designed to produce representative estimates of syphilis and HIV coinfection countrywide. At the second stage 14 households were randomly sampled within each selected village at first stage, except for Kigali city, where on average 14.5 households might be randomly sampled in selected villages. All eligible participants who consented to be interviewed and tested were included if they were residents or present in the sampled households on the night before the study. The sampling numbers were assigned sequentially within each village starting from one. The total number of households in the village was equal to the last serial number assigned. Due to the proportional allocation of the sample to the different strata, sampling weights were required to ensure that the sample is representative at the national level. A 95% CI, 80% power, design effect of 2, and assuming 20% lost to follow up during the 12-month period of follow-up, a national representative sample size of 13,810 adults was calculated using the prevalence of HIV. All selected participants at that moment filling inclusion criteria were consecutively enrolled in the survey.

### 2.2. Data Collection

A structured questionnaire was translated into Kinyarwanda and pretested in communities adjacent to those of the study population in a pilot process and back translated into English. All data collectors were trained according to their respective tasks on the administration of the questionnaire, blood sample taking procedures, ethical aspects specific to this survey, and processing the information provided by interviewees. Community health workers helped to identify villages and their boundaries as well as identify households and household members. The data collection tools were personal digital assistant (PDAs) in which answers provided by interviewees were entered. PDAs were synchronized to supervisors' computers and a checkup of questionnaire completeness and consistency was done at field level. After editing and correcting, data were sent to central level for management. A daily backup was done in each survey team.

### 2.3. Laboratory Data Collection

The syphilis screening test used rapid plasma reagin (RPR) tests (Macro-Vue RPR Card Test, BD, USA) to provide syphilis quick results to eligible participants. The RPR-positive samples were sent to National Reference Laboratory within 24 hours for confirmation using* Treponema pallidum* haemagglutination (TPHA) tests. The HIV rapid test was performed at the health center level in order to provide quick HIV test results to participants according to national testing guidelines. The blood sample was tested with three serial rapid tests according to national guidelines. For survey purpose, ELISA-based HIV testing was used. HIV Vironostika Uni Form I Ag/Ab, 4th generation, was used as a screening test. This was followed by Murex HIV antigen/antibody combination for confirmation of positive tests. If there was a discrepancy (i.e., Vironostika reactive and Murex nonreactive), the samples were confirmed by Enzygnost test. All samples with nonreactive results to Vironostika HIV Uni Form I Ag/Ab were considered negative.

### 2.4. Data Management and Analysis

Data were analyzed using STATA software (StataCorp LP, 4905 Lakeway Drive, College Station, TX, USA). To minimize data entry errors, a qualified data manager conducted data quality checks daily at site level. A second check was conducted at the central level within 5 days to allow data collectors to find respondents in case of errors identified in the quality checks. All variables, apart from provinces, were dichotomized to check potential association with main outcome variable, syphilis infection. Age was categorized in youth (aged 15–24 years) and adults. Univariate analyses were used to obtain summary statistics; bivariate analyses were used for cross-tabulation (chi-square), logistic regression, and estimating odds ratios of syphilis screening results. Multivariate analyses were used in logistic multiple regression to identify factors that collectively best predict syphilis infections. Factors that were associated with syphilis infection in the bivariate analysis at the ≤0.1 significance level were considered when computing the multivariable model. Model selection was conducted by minimizing the Akaike information criterion (AIC). Sampling weights reflecting the complex sampling design were used throughout the analyses.

### 2.5. Ethical Considerations

Participants meeting the inclusion criteria were recruited for HIV testing after signing a consent form. The survey protocol and all data collection tools were approved by the Rwandan National Ethics Committee.

## 3. Results

In total, 14,222 participants consented to participate in the survey and 14,140 people (99.4%) aged 15–49 years participated.  Table 1 provides the overall estimated prevalence within various strata.

The overall prevalence of syphilis using RPR screening tests was 0.9% (95% confidence interval (CI): 0.8–1.2). The overall prevalence of syphilis using the TPHA confirmatory test was also 0.9% (95% CI: 0.7–1.1). The prevalence of syphilis was higher in those aged 25–49 years (1.1%; 95% CI: 0.8–1.3) compared to those aged 15–24 years (0.6%; 95% CI: 0.4–0.9). The prevalence of syphilis was higher in respondents living in union compared to respondents not living in union (1.0%; 95% CI: 0.8–1.3) and (0.8%; 95% CI: 0.6–1.0). The prevalence of syphilis was similar in rural and urban residence with, respectively, 0.9% (95% CI: 0.7–1.1) and 1.1% (95% CI: 0.6–2.0). The lowest prevalence of syphilis was found in Northern Province (0.2%; 95% CI: 0.1–0.6); the highest was found in the city of Kigali (1.3%; 95% CI: 0.7–2.2), and the difference was statistically significant. Finally, respondents with secondary and higher education levels had a lower probability of being positive (0.4%; 95% CI: 0.2–0.8).

Overall, there was a slightly higher syphilis prevalence among women (1.0%; 95% CI: 0.8–1.3) than among men (0.8%; 95% CI: 0.6–1.0). The direction of this association, which is of having a slightly higher prevalence among women than men, was seen in all strata. Women in the Northern Province had a lower prevalence than men, as did women with higher education. The difference in syphilis prevalence between men and women was largest among non-Christians, where most of the increased syphilis among non-Christians (1.9%; 95% CI: 1.1–3.4) was observed in non-Christian women. Having said this, there were only 313 non-Christians among the 7,384 sampled women.

Table 2 displays the prevalence of syphilis infection among HIV-positive people and HIV-negative people.

The prevalence of syphilis among HIV-infected people was 6 times higher (4.8%; 95% CI: 2.9–7.9) than in HIV-negative study participants (0.8%; 95% CI: 0.6–1.0). This difference was statistically significant across all sociodemographic characteristics. For instance, in 15–24-year group, the prevalence of syphilis was 8.8 times greater in HIV-positive respondents compared to the prevalence of syphilis in the same age group of HIV-negatives. The prevalence of syphilis among males and females was significantly higher in HIV-positive compared to HIV-negative participants. Regarding residence, the prevalence of syphilis in urban residence was significantly higher (16 times) in HIV-positive participants compared to the prevalence of syphilis in HIV-negative participants. Even in the remaining sociodemographic characteristics (marital status, religions, education level, and wealth index), the prevalence of syphilis was significantly higher in HIV-positive study participants compared to HIV-negative study participants.

Table 3 summarizes the results of univariate and multivariate logistic regression analyses.

TPHA test results were considered as the dependent variable and age, sex, marital status, residence, religion, education level, wealth index, HIV status, and concurrent sexual partners in the 12 months preceding the survey were considered as independent variables. The strongest predictors of syphilis infection were being HIV-positive, which was associated with an adjusted odds ratio (aOR) of 4.2 (95% CI: 2.5–7.2), and respondents reporting that they had concurrent sexual partners in the last 12 months preceding the survey, which was associated with an aOR of 4.2 (95% CI: 2.8–6.5). Controlling for other factors, participants from other religions were associated with high prevalence of syphilis with an aOR of 1.9 (95% CI: 1.0–3.5). Other variables were associated with lower odds of syphilis prevalence: controlling for other factors, participants from southern province compared to the northern and participants with higher wealth index (4th and 5th quintiles) compared to low wealth index were associated with low prevalence of syphilis with aOR of 0.29 (95% CI: 0.11–0.76) and aOR of 0.44 (95% CI: 0.28–0.70), respectively.

## 4. Discussion

This household, population-based study conducted using a nationally representative sample showed that the overall prevalence of syphilis in Rwanda was 0.9%. It is the first time the prevalence of syphilis was assessed in the general population of Rwanda to inform prevention and treatment programmes. Our studies found various factors associated with increased probability of syphilis infection. This information will be critical for future campaigns to combat syphilis in Rwanda.

The Rwandan prevalence of syphilis was low compared to the 2.0% prevalence of syphilis found in India [13] and 3.5% among females and 3.9% among males in a population-based survey in the African region according to the WHO [1]. It was even lower compared to 1.8% of prevalence of syphilis found, respectively, in Kenya [10] and Uganda [14]. We assume that the reason of low prevalence of syphilis in Rwanda compared to other countries in the region is associated with the lower prevalence of HIV compared to the same countries [12]. These reasons have been hypothesized to include a national HIV programme, universal health care, and strong surveillance systems [15].

Our study found that the prevalence of syphilis was increasing with age from 0.6% in young people to 1.1% in older people. It was similar in Uganda, where the prevalence of syphilis increased from 1.2% in youth to 3.7% in older women and to 4.8% in older men [14], and in Kenya, where the prevalence of syphilis varied from 0.9% in young women to 2.5% in older women and it varied from 0.4% in young men to 4.4% in older men [10]. It was hypothesized that the prevalence of syphilis would be significantly higher in urban residences compared to rural residences, as is seen in HIV infection. But the prevalence of syphilis found in our study was not significantly different. As it turns out, this observation was similar to regional findings. For example, in Zambia, the prevalence of syphilis was the same, 4.2%, in rural and in urban [16]; in Kenya the observation was quite the same even when stratified by men and women [10]. Prevalence of syphilis in HIV-positive in Kigali (11.7%) was substantially higher than other provinces and on multivariate analysis. Syphilis and HIV have the same mode of transmission and the same risk factors. HIV prevalence was much higher in Kigali city (7.3%) compared to other provinces, for instance, Eastern province (2.1%) and it was threefold higher in urban settings compared to rural settings [12].

People with genital ulcers are vulnerable in acquiring and transmitting HIV [17]. In our study, the syphilis prevalence was much higher in HIV-positive respondents. In general, the prevalence of syphilis was 6 times higher in HIV-positive respondents compared to HIV-negative ones and across almost all sociodemographic characteristics (age, sex, marital status, residence, religion, education level, and wealth index). Syphilis is a treatable infection; targeting campaigns in HIV care facilities in a strong health care system such as Rwanda should bear impact and reduce overall disease incidence and ultimately prevalence.

The observed association between syphilis and religion, multiple concurrent partners, and wealth was all observed in similar settings. In Tanzanian rural population the traditional religion was also associated with syphilis infection (aOR = 1.6) [18] and syphilis infection was associated with having concurrent sexual partners (aOR = 1.8) [18]. In Kenya, researchers found that poorest/poorer and middle/richer were likely significantly associated with syphilis prevalence in males and females [10].

Our study has several strengths and limitations. The study design was robust with a large sample size across the whole of the nation. Through training, the use of experienced interviewers, and the use of multiple testing assays, the chance of measurement bias was minimized, which is often a risk in studies with large sample sizes. Nonetheless, some respondents may not have been comfortable to respond to sensitive questions related to sexual behaviour or sexual partners. This may have impacted data analysis, but we believe that these limitations did not significantly affect the final interpretation of study findings. Data collectors might have mistake in recording of respondents' responses.

In conclusion, data collected from this survey provided useful information to decision makers as baseline of prevalence of syphilis infection in the general population. Although data shows that the prevalence of syphilis was low, it is still persistent in Rwanda with the highest disease burden among HIV-positive individuals of reproductive age group and individuals reporting multiple sexual partners. To prevent HIV infection will significantly help in preventing syphilis and other STIs. The systematic screening of STIs should be reinforced especially among people living with HIV and youth.

## Figures and Tables

**Table 1:** Prevalence of syphilis by sociodemographic characteristics in Rwanda.

Demographic and socioeconomic characteristics	Women			Men			Total			
	RPR positive	TPHA positive		RPR positive	TPHA positive		RPR positive		TPHA positive	
	Percentage (95% CI)	*n*	Percentage (95% CI)	Percentage (95% CI)	*n*	Percentage (95% CI)	*n*	Percentage (95% CI)	*n*	Percentage (95% CI)
Overall 15–49	1.0 (0.8–1.3)	7,384	1.0 (0.8–1.3)	0.9 (0.7–1.2)	6,756	0.8 (0.6–1.0)	14,140	0.9 (0.8–1.2)	14,140	0.9 (0.7–1.1)
Age group										
15–24	0.7 (0.4–1.1)	3,008	0.8 (0.5–1.2)	0.6 (0.4–1.0)	2,612	0.5 (0.3–0.8)	5,620	0.6 (0.4–0.9)	5,620	0.6 (0.4–0.9)
25–49	1.2 (0.9–1.6)	4,376	1.1 (0.9–1.5)	1.1 (0.8–1.5)	4,144	0.9 (0.7–1.3)	8,520	1.1 (0.9–1.5)	8,520	1.1 (0.8–1.3)
Marital status										
Not living in union	0.9 (0.6–1.3)	3,657	1.0 (0.7–1.4)	0.6 (0.4–1.0)	3,192	0.6 (0.3–0.9)	6,849	0.8 (0.6–1.0)	6,849	0.8 (0.6–1.0)
Living in union	1.1 (0.8–1.5)	3,727	1.0 (0.7–1.4)	1.1 (0.8–1.5)	3,564	1.0 (0.7–1.4)	7,291	1.1 (0.9–1.4)	7,291	1.0 (0.8–1.3)
Residence										
Rural	0.9 (0.7–1.2)	6,507	0.9 (0.7–1.2)	0.8 (0.6–1.1)	5,854	0.7 (0.5–1.0)	12,361	0.9 (0.7–1.1)	12,361	0.9 (0.7–1.1)
Urban	1.5 (0.8–2.7)	877	1.3 (0.7–2.5)	1.1 (0.5–2.7)	902	0.9 (0.3–2.5)	1,779	1.3 (0.8–2.2)	1,779	1.1 (0.6–2.0)
Province										
East	1.2 (0.8–1.8)	1,652	1.2 (0.8–1.8)	0.9 (0.5–1.5)	1,590	0.8 (0.4–1.4)	3,242	1.0 (0.7–1.5)	3,242	1.0 (0.7–1.5)
North	0.2 (0.1–0.7)	1,239	0.1 (0.0–0.6)	0.4 (0.2–1.1)	1,064	0.4 (0.1–0.9)	2,303	0.3 (0.2–0.7)	2,303	0.2 (0.1–0.6)
South	0.7 (0.4–1.3)	1,848	0.8 (0.4–1.4)	0.9 (0.5–1.5)	1,725	0.9 (0.5–1.5)	3,573	0.8 (0.5–1.3)	3,573	0.8 (0.5–1.3)
West	1.5 (1.0–2.3)	1,755	1.5 (1.0–2.3)	0.9 (0.6–1.5)	1,432	0.6 (0.4–1.2)	3,187	1.2 (0.8–1.8)	3,187	1.1 (0.7–1.7)
City of Kigali	1.4 (0.8–2.6)	890	1.3 (0.7–2.5)	1.4 (0.7–2.9)	945	1.2 (0.5–2.7)	1,835	1.4 (0.8–2.4)	1,835	1.3 (0.7–2.2)
Religion										
Christian	0.9 (0.7–1.2)	7,071	0.9 (0.7–1.2)	0.9 (0.7–1.2)	6,324	0.8 (0.6–1.0)	13,395	0.9 (0.7–1.1)	13,395	0.8 (0.7–1.0)
Other religions	3.1 (1.6–5.8)	313	3.1 (1.6–5.8)	1.0 (0.4–2.6)	432	1.0 (0.4–2.6)	745	1.9 (1.1–3.4)	745	1.9 (1.1–3.4)
Highest education level										
No education/primary	1.2 (0.9–1.5)	5,878	1.2 (0.9–1.5)	1.0 (0.7–1.3)	5,235	0.9 (0.6–1.2)	11,113	1.1 (0.9–1.3)	11,113	1.0 (0.8–1.3)
Secondary/vocational/high education	0.4 (0.2–0.8)	1,506	0.4 (0.2–0.8)	0.6 (0.3–1.2)	1,521	0.5 (0.2–1.0)	3,027	0.5 (0.3–0.9)	3,027	0.4 (0.2–0.8)
Wealth index										
Lowest/secondary/middle	1.2 (0.9–1.6)	4,385	1.2 (0.9–1.6)	1.0 (0.8–1.4)	3,805	0.9 (0.6–1.3)	8,190	1.1 (0.9–1.4)	8,190	1.1 (0.8–1.4)
Fourth/higher	0.8 (0.5–1.2)	2,999	0.7 (0.4–1.1)	0.7 (0.4–1.2)	2,951	0.6 (0.3–1.1)	5,950	0.7 (0.5–1.1)	5,950	0.6 (0.4–1.0)

RPR: rapid plasma reagin; TPHA: *Treponema pallidum* haemagglutination.

**Table 2:** Syphilis infection and HIV status in Rwanda.

Demographic and socioeconomic characteristics	TPHA+ results among HIV+ participants aged 15–49		TPHA+ results among HIV− participants aged 15–49	
	*n*	Percentage (95% CI)	*n*	Percentage (95% CI)
Overall 15–49	412	4.8 (2.9–7.9)	13,728	0.8 (0.6–1.0)
Age group				
15–24	38	5.3 (1.3–19.8)	5,582	0.6 (0.4–0.9)
25–49	374	4.8 (2.9–7.8)	8,146	0.9 (0.7–1.1)
Sex				
Male	159	3.2 (1.3–7.6)	6,597	0.7 (0.5–1.0)
Female	253	5.9 (3.4–10.0)	7,131	0.8 (0.6–1.1)
Marital status				
Not living in union	150	3.5 (1.5–8.2)	6,699	0.7 (0.5–1.0)
Living in union	262	5.6 (3.2–9.7)	7,029	0.8 (0.6–1.1)
Residence				
Rural	320	3.3 (1.6–6.7)	12,041	0.8 (0.6–1.0)
Urban	92	9.8 (4.7–19.0)	1,687	0.6 (0.3–1.3)
Province				
East	72	4.3 (1.3–13.0)	3,170	0.9 (0.6–1.4)
North	44	2.2 (0.3–14.8)	2,259	0.2 (0.1–0.6)
South	95	1.9 (0.3–12.0)	3,478	0.8 (0.5–1.3)
West	97	1.2 (0.2–7.7)	3,090	1.1 (0.7–1.7)
City of Kigali	104	11.7 (6.1–21.1)	1,731	0.6 (0.3–1.2)
Religion				
Christian	366	3.6 (1.9–6.5)	13,029	0.8 (0.6–1.0)
Other religions	46	14.6 (6.0–31.4)	699	1.0 (0.5–2.0)
Highest education level				
No education/primary	339	5.6 (3.4–9.2)	10,774	0.9 (0.7–1.1)
Secondary/vocational/high education	412	1.2 (0.2–7.9)	2,954	0.4 (0.2–0.8)
Wealth index				
Lowest/secondary/middle	226	5.1 (2.6–9.9)	7,964	1.0 (0.7–1.2)
Fourth/higher	186	4.5 (2.3–8.6)	5,764	0.5 (0.3–0.8)

TPHA: *Treponema pallidum* haemagglutination; CI: confidence interval.

**Table 3:** Demographic and socioeconomic characteristics associated with confirmed syphilis infection in Rwanda.

Characteristics	Unadjusted model			Adjusted model		
	Odds ratio	*p* value	95% CI	Odds ratio	*p* value	95% CI
Age group						
15–24	1.0	—	—	1.0	—	—
25–49	1.7	0.008	1.15–2.52	0.8	0.461	0.52–1.35
Sex						
Female	1.0	—	—			
Male	1.3	0.227	0.87–1.78			
Marital status						
Living in union	1.0	—	—			
Not living in union	1.3	0.126	0.93–1.89			
Residence						
Urban						
Rural	1.3	0.249	0.82–2.15			
Province						
North	1.0	—	—	1.0	—	—
South	0.3	0.002	0.11–0.61	0.3	0.012	0.11–0.76
East	0.8	0.371	0.48–1.31	0.9	0.718	0.52–1.57
West	1.1	0.847	0.65–1.70	1.4	0.194	0.84–2.42
City of Kigali	1.2	0.441	0.72–2.11	1.7	0.091	0.92–3.31
Religion						
Christian	1.0	—	—	1.0	—	—
Other religion	2.3	0.004	1.31–4.02	1.9	0.038	1.04–3.48
Highest education level						
No education/primary	1.0	—	—	1.0	—	—
Secondary/vocational/high education	0.4	0.003	0.24–0.75	0.5	0.054	0.26–1.01
Wealth index						
Lowest/secondary/middle	1.0	—	—	1.0	—	—
Fourth/higher	0.6	0.009	0.41–0.88	0.4	0.001	0.28–0.70
HIV status						
Negative	1.0	—	—	1.0	—	—
Positive	6.2	<0.001	3.77–10.23	4.2	<0.001	2.46–7.23
Had concurrent sexual partners in the last 12 months						
No	1.0	—	—	1.0	—	—
Yes	3.8	<0.001	2.56–5.73	4.2	<0.001	2.79–6.46

CI: confidence interval.
